# Barriers and facilitators to adopting healthier lifestyle among low‐income women in Saudi Arabia: A qualitative study

**DOI:** 10.1111/hex.13735

**Published:** 2023-02-18

**Authors:** Samah Alageel, Maysa Alhujaili, Yasmin Altwaijri, Lisa Bilal, Reem Alsukait

**Affiliations:** ^1^ Community Health Sciences Department, College of Applied Medical Sciences King Saud University Riyadh Saudi Arabia; ^2^ Biostatistics, Epidemiology and Scientific Computing Department King Faisal Specialist Hospital and Research Centre Riyadh Saudi Arabia; ^3^ Alnahda Society Riyadh Saudi Arabia

**Keywords:** inequalities, lifestyle interventions, low income, qualitative research

## Abstract

**Introduction:**

Low socioeconomic status (SES) is significantly associated with increased levels of obesity, unhealthy diet, and physical inactivity leading to a higher risk of chronic diseases. This study aimed to explore low SES women's barriers and facilitators to engaging in a healthy lifestyle and their accounts in developing future behaviour change interventions.

**Methods:**

Qualitative study using focus group interviews informed by the Capability Opportunity Motivation—Behavior (COM‐B) framework. Interviews were conducted with a convenience sample, and data were analysed using thematic analysis. This study is conducted in partnership with Alnahda Society, a prominent nongovernmental organization in Riyadh, Saudi Arabia.

**Results:**

We conducted five focus groups with a total of 29 participants. We identified five overarching themes from the data related to participants' definition of a ‘healthy life’, the difficulties they face that hinder their engagement with a healthy lifestyle, the methods and reasons for changing health behaviour and participants' views of an ideal future behaviour change intervention. Women's definition of a healthy lifestyle did not only include a healthy diet and physical activity but also emphasized the importance of improving mental wellness. Following a healthy lifestyle, although desired, is not always a priority for women with low SES due to the high cost, lack of availability of healthy options and time constraints. Many women in our sample discussed the need to have a routine and discipline to follow and maintain a healthy lifestyle. Family members' support for behaviour change was discussed as a facilitator to maintaining a healthy lifestyle. Women highlighted several reasons that would motivate them to change their health behaviour, including having or preventing health conditions, improving mental health, and managing weight. Participants also discussed the characteristics of an ideal behaviour change intervention.

**Discussion:**

This study suggests that women with low SES are faced with several barriers to adopting a healthy lifestyle. Behaviour change intervention targeting this population needs to be tailored to address these barriers and facilitate behaviour change for people with limited resources. National policies to improve the availability and affordability of healthy options are also needed to reduce health disparities.

**Patient and Public Contributions:**

Women of low SES who took part in the study were given a chance at the end of each focus group discussion to reflect on the questions and add any areas important to them that were not covered during the interview. Experts working with disadvantaged populations in a nonprofit organization (Alnahda society) contributed to the design of the topic guide.

## 1 INTRODUCTION

Noncommunicable diseases (NCDs) are the main driver of morbidity and mortality in Saudi Arabia, accounting for 73.2% of deaths.^1^ As the Saudi population ages, the disease burden is likely to increase, emphasizing the need for targeted community‐based interventions to help prevent and reduce the prevalence of NCDs. Women in Saudi Arabia have substantially higher rates of risk factors that lead to NCDs than men. For example, the prevalence of overweight and obesity among women in Saudi Arabia is 54%, with 82% of women not meeting physical activity recommendations and only 7% of women consuming the recommended fruits and vegetable servings.^2^ Additionally, a recent analysis of NCD‐related socioeconomic inequalities in Saudi Arabia showed that the prevalence of NCDs is highest among women with lower income and education levels.^3^ These findings highlighted the need to develop interventions for this high‐priority target group to reduce health inequality.

Community‐based interventions targeting low socioeconomic status (SES) groups are lacking in Saudi Arabia, despite their high potential for scalability, impact, and cost‐effectiveness using limited resources.^4^, ^5^ A systematic review of community‐based interventions targeting dietary behaviour and physical activity in low SES groups suggested that many barriers and facilitators to behaviour change were not addressed by current interventions.^6^ The review suggested that many deeper social, psychological and practical barriers associated with engaging in healthy behaviour were not addressed, likely reducing the effectiveness of these interventions.

Socioeconomic position is likely to influence people's perceptions of the importance of a healthy lifestyle and factors that influence adopting healthy behaviour. It has been suggested that low SES groups are more concerned about the costs of healthy behaviour and are more motivated to change as a result of a health condition rather than to prevent diseases.^7^ A qualitative study with residents of low‐income communities suggested that although participants in their study had a high level of knowledge of what constitutes a healthy diet, barriers to healthful eating included high costs and lack of availability of healthy stores in their neighbourhoods.^8^ Concerns over neighbourhood safety and lack of time and energy were more commonly reported barriers to physical activity among low SES groups.^7^ Previous studies suggested that providing social support and tailored interventions in groups of like‐minded companions seemed to facilitate change among low SES groups.^7^, ^9^, ^10^ However, most evidence is conducted in developed countries, and less is known about factors that influence behaviour and facilitate change among low SES individuals in Saudi Arabia, specifically women. Accordingly, we aimed to investigate low SES women's barriers and facilitators to engaging in healthy lifestyles and to explore their accounts in developing behaviour change interventions.

## 2 METHODS

### 2.1 Study design

The study employed a qualitative research approach, using focus group interviews with women to explore barriers and facilitators to engaging in a healthy lifestyle and their accounts in developing behaviour change interventions. A healthy lifestyle was defined in relation to a healthy diet and physical activity. However, participants' definitions of ‘healthy lifestyle’ was explored to consider the important aspects of healthy lifestyles beyond diet and physical activity from the participants' views. This study was conducted in partnership with the Alnahda Society.

### 2.2 Study setting, sampling and recruitment

Interviews were conducted with Alnahda beneficiaries using convenience sampling. Alnahda staff introduced the study to Alnahda beneficiaries, and the research team contacted potential participants who were interested to join the study. We determined the sample size and the number of focus group interviews through inductive thematic saturation. Thematic saturation was determined when no new codes or themes emerged.^11^

Alnahda Society is a nonprofit organization dedicated to empowering women socially and economically through the execution of numerous projects and programs in Saudi Arabia. Alnahda Society works to achieve its mission through three main workstreams: local development, research and advocacy. Local development aims to assist low‐income households to graduate out of poverty. Financial support aims to target unmet needs through direct cash transfers, monthly food stamps, transportation, and so forth. Career education and capacity development support aim to equip beneficiaries with skills to become active contributors to the development of Saudi society. Households eligible to benefit from Alnahda's support are those with a monthly income of less than 1000 Saudi Riyals (equivalent to 266 US dollars) per family member. On average, beneficiary households consist of six members and earn an average monthly income of 2550 Saudi Riyals (equivalent to 678 US dollars). In 2021, Alnahda Society supported 340 families, 59% of which were headed by women, and provided all families with monthly food support and over 200 families with housing support.^12^

### 2.3 Interview guide

The interview guide (Supporting Information: Appendix 1) was developed following the literature and theories of health behaviour change.

In an attempt to combine theories of behaviour change and produce a comprehensive model, Michie et al.^13^ have developed the Capability, Opportunity, Motivation—Behavior (COM‐B) model of behaviour change. The COM‐B model suggests that in order for a behaviour to change, change is required in at least one of the following components: an Individual's capability to carry out the behaviour, the opportunity for the behaviour to occur, and the motivation to perform the behaviour.^13^ The framework is intended to be comprehensive and applicable to different health behaviours. The use of the COM‐B framework helps in identifying a variety of perceived factors that influence health behaviour to develop effective interventions that are responsive to the target group. COM‐B has been widely applied to explore barriers and facilitators to a healthy diet and physical activity, including studies focusing on low‐income groups.^14^, ^15^, ^16^ We used the COM‐B model to explore participants' lifestyles and their perceptions of barriers and facilitators of healthy eating and physical activity, including social influences. We discussed views on future health promotion interventions as well. Participants also had the freedom to discuss any relevant areas that were not covered by the guide. The topic guide was shared with experts working with disadvantaged populations in Alnahda Society for their input and contributions. Experts' feedback confirmed the relevance of the questions to the target group and suggested rephrasing some of the questions for suitability and encouragement of the discussion. All changes were made before the start of the data collection.

### 2.4 Interview procedure

All participants gave informed consent to participate in the study before the focus group interviews. Before the interview, a short questionnaire was used to collect sociodemographic data, including age, marital status, employment status, and education level. Interviews were conducted in November 2021, and each interview lasted approximately an hour. Interviews were conducted virtually due to the global pandemic caused by COVID‐19. All interviews were conducted in Arabic, and relevant quotes were translated into English by the authors. Each focus group interview consisted of five to eight participants. Focus group interviews were facilitated by two members of the research team (S. A. and M. A.). S. A. is a Saudi, female public health researcher experienced in qualitative research. M. A. is a Saudi female clinical dietitian with training in qualitative research. Both researchers did not have any prior connections to the study participants.

### 2.5 Data analysis

The interviews were audio recorded and transcribed verbatim. A reflexive thematic analysis was conducted.^17^ As part of the familiarization stage, two authors (S. A. and M. A.) read through the transcripts before coding, where initial impressions and possible themes were created. S. A. and M. A. independently coded all focus group interviews using ATLAS.ti software. Codes were then compared, revised, then discussed with R. A., who is a Saudi female researcher in food and nutrition policies and programs. Any necessary amendments were made until full agreement among research members was reached. An inductive approach to coding was applied, where codes and themes were generated from the data. The relationship between codes was then examined and discussed with the whole research team to develop preliminary themes. Themes are then assessed and refined iteratively to ensure a comprehensive interpretation of the data in relation to research objectives.

## 3 RESULTS

We have conducted five focus group interviews with a total of 29 participants. The sample included women from different ranges of age groups and marital statuses (see Table 1). The majority of participants were 31–40 years old and had a secondary education degree. We have identified five overarching themes from the data.

**Table 1:** Study participants characteristics.

Age	18–30 years old	2
	31–40 years old	20
	41–50 years old	7
Educational level	Below intermediate education	9
	Intermediate education	6
	Secondary education	11
	Bachelor and above	3
Employment	Employed	6
	Unemployed	23
Marital status	Single	1
	Married	10
	Divorced	15
	Widowed	3
Has children	Yes	28
	No	1

### 3.1 Theme 1: Definition of a ‘healthy life’

Participants discussed the way they define a healthy lifestyle, mainly pertaining to a healthy diet and physical activity. The definition of a healthy diet ranged from eating more fruits and vegetables to eating at home prepared meals rather than takeout. Some participants defined a healthy diet as minimizing the use of oil and sugar and increasing dairy product and legume consumption. Many participants also mentioned that a healthy diet meant cutting down on soft drinks and substituting them with fresh fruit juice.

I have a system here, to choose what is useful for the body, because I take care of my elderly parents, so I manage our diets according to their needs. A diet low in sugar, we use little salt, no oil. We rarely fry, we do not have soft drinks, mostly fresh juices. (Focus group 1, P3, 31–40 years old, Employed)

The definition of physical activity was mainly linked to participants' daily routines. Many participants defined physical activity as walking. Others also viewed physical activity in relation to housework, such as tidying up and cleaning the house. Some participants linked physical activity to taking care of their children or elderly family members.

Such as cleaning, arranging the house, and taking care of my elderly parents, this is my activity in the house. (Focus group 1, P1, 31–40 years old, Unemployed)

Many participants highlighted the importance of mental health as part of a healthy life. Stress, anxiety, and overthinking were among the main reasons for focusing on promoting mental health. Some participants discussed mental health as the core of overall health, therefore it must be nurtured and preserved.

I feel if mental health improves, the rest of your health will improve. (Focus group 3, P6, 31–40 years old, Employed)

Some participants highlighted the social health aspect of a healthy life. Having healthy relationships with people was linked to an overall healthy life.

I expect a healthy life is related to all the healthy things, in terms of eating and drinking. Physical, psychological, and social relations, in all respects. (Focus group 2, P3, 41–50 years old, Unemployed)

Some participants discussed the role of sleep as part of a healthy lifestyle. Participants shared their experiences with poor sleep patterns and discussed how they affected their mental and physical health negatively.

In terms of work, my shift was in the evening, and everything turned upside down, my sleep and eating habits, and it affected my psyche and the psyche of my family. Until I turned to the morning shift, my life changed completely. (Focus group 5, P1, 31–40 years old, Employed)

#### 3.1.1 Sources of health information

Participants' definitions of a healthy lifestyle were influenced by the sources of health information they relied on. The media and the Internet were the main sources of health‐related information. Following health experts in nutrition and psychologists in the media was often cited as a way of being up‐to‐date with health information. For some participants, peers were the main source of health information.

From the television, the people I follow on snapchat, nutritionists, self‐development and psychologists. … I also turn to my neighbors for information. (Focus group 5, P2, 41–50 years old, Unemployed)

Some participants were concerned with the accuracy of health information they read on the Internet. Others felt confused and unsure of the advice they receive from social media as, in some instances, it seemed contradictory and unclear. Participants discussed how healthcare professionals' advice may be subjective and unrealistic, making it difficult to adopt the health advice.

Google sometimes provides you with answers that are not accurate. I follow nutritionists. They confuse me more…. They provide different advice. I mean, I follow [name of a nutritionist], she always says, ‘stop eating eggs, stop drinking milk’. If I get up in the morning and I intend to eat healthy food, I am confused, I do not know whether to eat these things or not. (Focus group 2, P2, 41–50 years old, Unemployed)

### 3.2 Theme 2: ‘Life is difficult’, struggling to follow a healthy lifestyle

Participants discussed the difficulty of following a healthy lifestyle due to the many daily constraints. A healthy lifestyle is often viewed as a luxury that not everyone can afford.

Certainly, it is not an easy thing, when one says a healthy life, it means free of problems, free of poverty, it means many things, not anyone can reach a healthy life. (Focus group 2, P3, 41–50 years old, Unemployed)

#### 3.2.1 Cost and availability

Cost is one of the main barriers to healthy living among participants of this study. Participants struggled with limited financial resources, which impact their health in two main ways. The financial difficulty had a negative impact on their mental health due to worrying about their inability to provide basic life necessities.

If someone is financially well, they can be comfortable, they no longer worry about anything, everything would be fine… money plays a role, affects human health, you know, it tires you psychologically. (Focus group 2, P5, 31–40 years old, Unemployed)

Participants mentioned cost as a reason for their inability to afford healthy food options. Participants acknowledged the importance of a healthy diet but were often constrained by their limited finances. Some participants discussed this in relation to the time they received their financial aid.

Well… it depends on the circumstances, things are different at the beginning of the month, then it changes in the middle or at the end of the month (laugh)… The finances at the first of the month are different from the end of the month. At the end of the month, you would eat from whatever is available. (Focus group 2, P4, 31–40 years old, Unemployed)

Others explained that their food choices were highly dependent on cost. They explained that the basis for their choices is lower cost rather than any other reason.

We buy the cheapest rather than the healthiest things. (Focus group 4, P7, 41–50 years old, Employed)

Some participants explained ways of mitigating the impact of their limited income on their food choices. For example, using food coupons offers or borrowing money.

I buy food every Monday, there is an offer ‘buy a kilo and get a kilo for free’, we eat a kilo and put a kilo in the fridge. (Focus group 2, P2, 41–50 years old, Unemployed)

The availability and ease of access to healthy options were also discussed as a barrier to a healthy lifestyle. Some participants discussed their inability to exercise due to the lack of suitable places to walk within their neighbourhoods, especially for women.

Not all neighborhoods have a suitable walkway for women to walk. (Focus group 1, P4, 31–40 years old, Employed)

Participants discussed their preference for walking as a form of exercise rather than going to the gym due to cost and the unavailability of suitable places. Although many participants expressed many perceived benefits of going to gyms; the high costs of gym memberships made it impossible for them to join. Furthermore, some participants dreaded the additional cost of transportation, therefore they preferred home‐based physical activities.

For me, I would like to join a gym, but the prices are exaggerated, it really does not suit us. Like my situation, I have no income except for the financial aid, or the benefits from the association, my budget is restricted. (Focus group 4, P5, 41–50 years old, Unemployed)

#### 3.2.2 Time and work

Time constraint was often mentioned as another major barrier to a healthy lifestyle. Participants did not have the time to prepare healthy meals and engage in physical activities. Many participants discussed the difficulty of managing the time needed to adopt a healthy lifestyle, especially with their stressful lives.

I mean, time is the main reason. Also, possibly because of my work, many things, I can only say Alhamdulillah [thank God], these are my circumstances. So, it is mainly time and work. A pressure from the two. (Focus group 4, P3, 31–40 years old, Unemployed)

Differences were observed among participants based on employment status. For employed participants, time was the most significant barrier to following a healthy lifestyle. While unemployed participants mentioned cost as the main barrier, followed by time constraints.

#### 3.2.3 Social pressure and responsibilities

Many participants discussed the difficulty of following a healthy lifestyle as a result of their life pressures and responsibilities. Most participants were the sole or main providers for their families, which served as a constant source of stress. Social pressure included taking care of their children and elderly parents. These life stressors had a negative effect on their mental health, further contributing to the difficulty of adopting a healthier lifestyle.

As for me, I don't have time, I come back from work to attend to the needs of my parents and my children. If my parents want something from outside, because I don't have siblings, I bring it, sometimes I fall asleep without even knowing I did. (Focus group 1, P3, 31–40 years old, Employed)

### 3.3 Theme 3: How to change health behaviour?

#### 3.3.1 Change needs routine and discipline

Many participants discussed that lifestyle change requires developing a routine. Developing a routine, although challenging at the beginning, helps in maintaining lifestyle changes. Changing health behaviours requires discipline to break old habits and a willingness to persist with these changes.

We are used to eating and sitting, we don't walk anymore. To burn fat after eating it requires time and effort… But one must develop a system so that change lasts. (Focus group 1, P1, 31–40 years old, Unemployed)

As participants linked the ability to change with being disciplined, lack of change was often viewed as ‘laziness’. Participants referred to themselves as ‘lazy’ when unable to change or unable to follow through and maintain changes in their lifestyle.

I don't know why I get excited at the beginning and then stop. Maybe because my will is weak. (Focus group 5, P3, 31–40 years old, Employed)

#### 3.3.2 Family members as a facilitator and a barrier

Most participants discussed the role of their families in facilitating change to a healthy lifestyle, as they perceived these changes to be made on a family level. For many participants, their families acted as a barrier, mainly resisting changes in the family's dietary habits. The younger generation's resistance to a healthy lifestyle stems from the perceived lack of short‐term negative impact of an unhealthy lifestyle. A healthy diet was perceived to be a necessity for the elderly or for those with medical health problems.

I mean, my children tell me, ‘Leave us to enjoy life mom. Why are you feeding us old people's diet? Do not eat! do not do that! we eat and walk’. I mean, in the long run, these things will harm them, salt, sugar, or excessive spices. (Focus group 5, P1, 31–40 years old, Employed)

A healthy diet was often viewed as being less flavourful and therefore, not always preferred or accepted by participants' families. Many participants discussed trying different recipes to improve the flavour of the food while attempting to preserve health benefits. Participants reported that their families' initial resistance to changes was often followed by acceptance.

My husband prevents me from dieting, and he is stubborn, he gets upset, and he does not like healthy eating, although I try to master it, but he does not like the healthy lifestyle. But after a while he knew that this was my lifestyle and I am committed to it and got used to it, and even my kids got used to it. (Focus group 1, P2, 41–50 years old, Unemployed)

Some participants reported a positive impact of their families on their healthy lifestyles. Family members can encourage and facilitate health behaviour change. The engagement of family members in the process of lifestyle change is often viewed as making behaviour change easier and even more enjoyable.

I have my daughters and my husband. If they see me sitting down, they say ‘Mom, let's do a physical activity’. When I come home from work and I'm tired, they say we go for a walk now then eat. (Focus group 4, P7, 41–50 years old, Employed)

### 3.4 Theme 4: Why change health behaviours?

All participants believed in the need to change their unhealthy behaviours and follow a healthy lifestyle. Several motivations to change health behaviour were mentioned by the participants, either by experience or perceived as a benefit of changing their lifestyle.

#### 3.4.1 Health conditions

Many participants stated that their motivation to improve their health behaviour was a recent medical diagnosis. The diagnosis served as a wake‐up call to change their lifestyle and improve health outcomes. These medical conditions were either experienced by the participants themselves or by a family member, motivating the need to improve their overall health.

When I go to a medical follow‐up, and they check my blood sugar. They tell me that you are on the edge of becoming diabetic, so it gives me an indication of self‐awakening, ‘cut off sugar’. (Focus group 1, P4, 31–40 years old, Employed)

Mothers in the sample were often motivated to improve their health behaviour to promote their children's health.

The health of the children, they are weak or have diseases, we need to keep them away from unhealthy food. (Focus group 3, P5, 41–50 years old, Unemployed)

For participants with existing health conditions, experiencing an improvement in their symptoms acted as an incentive to maintain healthy changes. Other participants were motivated to change their health behaviours to avoid future health complications

If you see changes in your body, you will continue running without even thinking. (Focus group 3, P8, 31–40 years old, Unemployed)

#### 3.4.2 Weight management

Participants' experiences of weight loss were often cited as a motivator to change and maintain healthy behaviours. Weight gain was alarming for some participants, promoting changes in their lifestyles, especially if accompanied by comments on their weight from family or friends.

We were at a family gathering and one of the ladies got the weight scale, and I was the heaviest one of them. One of them laughed at me saying that the scale will break. I went home and intended to start exercising. I signed up for a program and received messages on the mobile, for example, drinks that contain sugar increase weight and herbs that help lose weight. (Focus group 1, P3, 31–40 years old, Employed)

#### 3.4.3 Energy and strength

Some participants believed that following a healthy lifestyle leads to having more energy in their daily life. Being physically active and having a healthy diet were perceived to reduce fatigue and improve overall physical strength.

When I was living with my husband's family, they used to eat fried food and soft drinks and I was always tired. After we separated, I went to my family's home, there was no fried food or soft drinks, and I felt an abnormal energy. (Focus group 1, P1, 31–40 years old, Unemployed)

#### 3.4.4 Mental health

Most participants believed that a healthy lifestyle leads to improvements in mental health. Changes in diet, physical activity, and sleep patterns were believed to promote mental health. Physical activity was believed to be the most important influencer on mental health. Many participants were motivated to walk regularly as a result of the mental health benefits they experienced.

I sometimes have negative energy because I have problems with my ex‐husband, I am nervous, I am depressed, I feel that I don't want to talk to anyone. And I go for a walk, it clears my head, I feel a little better when I go for a walk, for an hour or two hours, all the negative energy is substituted with positive energy. (Focus group 5, P4, 31–40 years old, Unemployed)

### 3.5 Theme 5: Ideal behaviour change intervention

Participants explained their views of an ideal health behaviour change intervention, including information and skills needed, mode of delivery, and form of education.

#### 3.5.1 Needed health information and skills

Participants mentioned examples of health information they need to follow a healthier lifestyle. Some participants perceived themselves to have many misconceptions and were concerned about their inability to distinguish between reliable and inaccurate health information. Some participants expressed their inability to identify and define a ‘healthy meal’. They lacked an easy and fast way to get nutrition‐related health information.

Sometimes we have a misunderstanding about calories, the amount of fat and sodium, in addition to WhatsApp rumors, everyone gives wrong information, like if you eat a lot of zucchini, your sugar will decrease. (Focus group 1, P4, 31–40 years old, Employed)

Participants reported that they were unable to identify the ‘right’ amount of physical activity they need. Physical activity experts were believed to be necessary to provide advice on the most suitable activity type and level for them, depending on their health status.

I am worried that I don't know what is useful and what is harmful… for example those who have joints or knees injury, cannot do some types of exercises, so you work with someone who knows your health condition and provides you the appropriate training. (Focus group 3, P4, 31–40 years old, Unemployed)

Some participants requested to be educated on reading the product information on food labels to understand the food product's content. They also highlighted the need for Arabic labels.

For me, it is difficult to read the ingredients of the product, sometimes it is required from the Ministry of Commerce or the Food and Drug Authority to translate food labels, but the exact details of the product are not all translated. (Focus group 1, P4, 31–40 years old, Employed)

Many participants discussed the need for mental health support in the program. Participants expressed the need to learn the skills to manage their stress and control their emotions. Some participants asked for counselling on how to deal with their children to optimize their mental health.

Psychological health. Attention to mental health is nice. For me, I want to manage my nervousness. (Focus group 5, P1, 31–40 years old, Employed)

#### 3.5.2 Mode of delivery

Participants discussed possible modes for delivering a health promotion program. Face‐to‐face and virtual modes of delivery were discussed and compared. Many participants preferred face‐to‐face interventions as it was seen as a form of socialization with peers. Social contact was believed to have several benefits, including entertainment and improving mental health.

When we are together, we laugh and we chat with each other, so that mentally it is better. (Focus group 4, P1, 31–40 years old, Unemployed)

Some participants justified their preference for a face‐to‐face intervention as it improves comprehension of the program content and it promotes engagement. These views were supported by the participants' previous experiences with virtual and face‐to‐face programs.

Face to face is better than after … I understand you more. I am more focused when it is face to face than virtually. (Focus group 5, P3, 31–40 years old, Employed)

Participants' preference for virtual sessions was due to their competing responsibilities limiting their free time. Some participants cited transportation as a barrier to attending face‐to‐face programs as well. If transportation was not an issue, many participants prefer face‐to‐face over virtual sessions.

Yes, it [face‐to‐face sessions] is an obstacle for mothers who have young children. Yes, it is an obstacle as no one will take you there. (Focus group 4, P5, 41–50 years old, Unemployed)

#### 3.5.3 Form of education

Participants discussed whether the program should be conducted one‐to‐one or offered in a group setting. Many participants preferred group sessions as they would provide peer support. Some participants also mentioned that attending health promotion programs in groups would enhance learning as people would learn from the experience of others. Participants mentioned that group sessions would enhance their engagement with the program and help them connect with program providers.

In groups would be nice … Because of the participation of each member, each one would express her opinion. It would increase enthusiasm with the program…. Sharing opinions will be nice. We take ideas from each other and benefit from the experiences of the other women. (Focus group 5, P5, 31–40 years old, Unemployed)

Some participants proposed engaging family members in the program. Providing the program for mothers and their children was perceived as a facilitator to engagement, enhancing the participants' experience.

Nice, so that I can come with an Uber, me and my daughter. If the program is for groups and in the afternoon and provides exercises after we finish our work and schools, it would be great! (Focus group 5, P2, 41–50 years old, Unemployed)

Other participants preferred one‐to‐one sessions as they provided the privacy needed to discuss sensitive subjects difficult to discuss in group settings. Others emphasized the importance of personalization in health promotion messages, which can only be offered in one‐to‐one consultations. Some participants explained that when health advice is offered in a group setting it could be confusing and would not fit all.

If it was an individual, it would have its benefits, like things that are not applied to everyone… for example the things you would say to a psychiatrist you wouldn't even say in front of your own children… As for groups, you would only share general information. (Focus group 5, P5, 31–40 years old, Unemployed)

## 4 DISCUSSION

This study aimed to explore barriers and facilitators to a healthy lifestyle among women with low SES in Saudi Arabia and their accounts in developing behaviour change interventions. Women's definition of a healthy lifestyle did not only include a healthy diet and physical activity, but it also emphasized the importance of improving mental wellness. Following a healthy lifestyle, although desired, was not always a priority for women with low SES. This was due to the high cost of a healthy lifestyle, lack of availability of healthy dietary options, and time constraints. Findings from this study suggest that following a healthy lifestyle is highly influenced by both social and physical opportunities. While participants in our study had the motivation to improve their lifestyles, change is hindered by women's capability (knowledge and skills) and opportunity (environmental influences and social support).

Women in our study mentioned that their healthy lifestyle choices were restricted by their limited resources. A study among low‐income elderly populations suggested that the high cost and the lack of availability of healthy food options were key barriers to adopting a healthy diet.^18^ Low self‐efficacy to change health habits and difficulty accessing healthy food due to lack of transportation were also reported as barriers.^18^ A systematic review of factors affecting the uptake and maintenance of health behaviour suggested that lack of time, social responsibilities, work pressures, and financial costs are among the most common barriers to changing health behaviours.^19^ A meta‐ethnographic study aimed at assessing factors that influence healthy eating suggested that although affordability and availability of healthy food affect people's diet regardless of their SES, their impact is more pronounced in low SES groups.^20^

Family played a major role in women's ability to follow a healthy lifestyle. Women in our study discussed how family members could motivate them to be more physically active or introduce improvements in their diet. While others explained that lack of family support could hinder health change. The English Longitudinal Study of Aging suggested that people are more likely to follow positive health behaviour changes if their partners join in adopting these positive changes.^21^ Evidence from several studies suggested that family and friends' support was a determinant factor in engaging in healthy dietary behaviour and physical activity.^19^ Parents are incentivized to follow healthy lifestyles to act as good role models for their children.^19^ This was consistent with our findings, where women's motivation to change their health behaviour stemmed from their desire to improve their children's health.

Women in our study suggested including family members as a part of the proposed behaviour change intervention. It was viewed as improving engagement and as an entertaining aspect of the program. A study on low‐income families suggested that positive changes to a healthy lifestyle were more sustainable when all family members were involved in making these changes.^22^ A recent systematic review suggested that physical activity and mother‐daughter interventions had the potential to increase health behaviour among both mothers and daughters.^23^ These results emphasize the potential benefit of involving family members in behaviour change intervention as it may facilitate the initiation and maintenance of health behaviour change.

Our study participants also suggested that future behaviour change interventions could be provided in the form of face‐to‐face group sessions, which would motivate participation in the intervention as it would add the social element that the participants valued. A review of community‐based interventions in low‐income communities in the United Kingdom suggests that providing socially inclusive and enjoyable activities can improve program acceptability.^6^ Group sessions were viewed as providing personal connections with peers, which acted as a motivating factor for continuous engagement with behaviour change programs.^9^ Although most women in our study preferred future behaviour change intervention to be provided face‐to‐face, virtual interventions were seen to have some potential benefits. Ease of access and continuation were cited as potential benefits of virtual intervention. While face‐to‐face interventions provide human support that has been associated with increased effectiveness and adherence, it has been suggested that virtual intervention could provide a virtual person‐to‐person component, potentially substituting in‐person interventions.^24^ With the increase in the popularity and potential effectiveness of digital intervention, there is a need to consider disparities in relation to access, proficiency, and skills needed to effectively participate in these interventions, especially in older adults and low‐income populations.^25^

### 4.1 Future research and policy implications

There is a need to prioritize interventions targeting barriers to healthy lifestyles among people with low SES. Interventions could include strategies that help women integrate health behaviour change into their daily routines, including skills to prepare affordable home‐cooked meals and home‐based exercise. Offering counselling on budgeting and time management could also be beneficial to facilitate behaviour change. Our findings highlighted that the social component should be considered in future behaviour change interventions. It has the potential to provide mental health benefits and could facilitate participant engagement in the program.

Although women in this study provided suggestions for potential behaviour change interventions, many of the barriers to healthy living need to be addressed on a policy level. The collaborative effort of different governmental agencies and health sectors is necessary for effective health behaviour change interventions. There is a need for national policies and programs to improve the availability, accessibility, and affordability of healthy living (i.e., healthy foods, fitness centres, and wellness programs). For example, providing accessible, inclusive, and low‐cost facilities for physical activity. Providing an environment conducive to physical activity, in addition to improving the quality and prices of dietary choices, has the potential to promote behaviour change. Additionally, primary care centres and general practitioners need to have an active role in encouraging and promoting behaviour change.

Existing literature on the barriers and facilitators of healthy lifestyles and effective behaviour change interventions is predominantly focused on developed countries and affluent populations. Health intervention research among disadvantaged populations and in developing countries is lacking. There is a need to produce evidence‐based literature on factors influencing health behaviour and effective interventions in other settings, especially among people with lower SES.

### 4.2 Strength and limitations

This study, to the authors' knowledge, is the first study to examine barriers and facilitators to a healthy lifestyle among women with low SES in Saudi Arabia. A strength of this study is that it included women from different age groups, marital statuses, and employment statuses. This has helped in accessing a comprehensive view of women's experiences and factors that impact their lifestyles. Using the COM‐B model may have led to participants discussing factors that may not be otherwise discussed. The model provides a comprehensive framework that includes different behavioural influences.^13^ Focus group interviews provided the chance to understand people's views and experiences in a naturalistic setting, where social context is more influential than individual accounts.^26^ As many views and perceptions naturally emerged through these discussions, researchers' influences on the interview are believed to be minimized. Additionally, all interview transcripts were independently coded by two researchers, which improved the rigour and credibility of interpretations.^27^

This study is not without limitations. Due to data confidentiality, the participants in this research were contacted by Alnahda staff, who contacted and recruited the participants for the interviews. This may have introduced a bias, as those who were not recruited or were unable to participate in the study may have had different experiences. Qualitative interviews discussing healthy behaviour can produce social desirability bias, especially if conducted virtually.^28^ The interviewers were external to Alnahda Society, with no conflicting roles or affiliations, which is believed to help in accessing more private accounts and reducing socially desirable responses. Online focus groups have several limitations compared to face‐to‐face interviews, including a lack of control over the interview environment in addition to technical difficulties and connection issues.^29^ Participants could be distracted from the interview by engaging with family members or colleagues. The interviewers tried to encourage the engagement of all participants by calling them by their names. Participants with technical problems were offered to reschedule the interview.

## 5 CONCLUSION

This study suggests that women with low SES face several barriers to adopting a healthy lifestyle. Behaviour change intervention targeting disadvantaged populations needs to be tailored to address the specific barriers and facilitators to promoting behaviour change for people with limited resources. National policies to improve the availability and affordability of healthy options are highly needed to reduce health disparities.

## AUTHOR CONTRIBUTIONS

Samah Alageel, Reem Alsukait, Lisa Bilal and Yasmin Altwaijri contributed to the conceptualization and design of this project. Interviews were conducted by Samah Alageel and Maysa Alhujaili, and data analysis was conducted by Samah Alageel, Maysa Alhujaili and Reem Alsukait. Emerging results were discussed with all the authors during regular meetings. Samah Alageel and Maysa Alhujaili wrote the first draft of the paper; all authors contributed to reviewing and editing subsequent drafts and reviewed the final manuscript.

## CONFLICT OF INTEREST STATEMENT

The authors declare no conflict of interest.

## ETHICS STATEMENT

This study was approved by the Research Advisory Council (RAC) of King Faisal Specialist Hospital and Research Center (RAC # 2211040).

## Supporting information

Supporting information.

## Data Availability

The data are not publicly available due to privacy and ethical restrictions.
