# Supplementary material for: Barriers and facilitators to adopting healthier lifestyle among low‐income women in Saudi Arabia: A qualitative study
Source: Health Expect. 2023 Feb 18;26(3):1202–12. doi: 10.1111/hex.13735 (PMC10154786; doi:10.1111/hex.13735)
Supplement: Supplementary file 1 — Supporting information. [file HEX-26--s001.docx]

**Appendix 1: Topic guide**

**Overall questions:**

Can you explain what you understand by a “healthy lifestyle”? **(Knowledge – which is a form of capability)**

**Probes:** What comes to mind when you hear the term “healthy lifestyle”?

How do you define a healthy lifestyle?

Is following a healthy lifestyle a concern to you? **(Capability)**

Do you think following a “healthy lifestyle is time consuming? Not affordable? **(Opportunity – Physical and social)**

**Diet related questions:**

Describe what healthy eating means to you.

**Probes:** How would you describe a ‘healthy meal’? How do you decide if a food is good for health? What foods do you think are good for health? **(Knowledge-perceptions)**

Describe how you decide what foods to eat/ plan your diet. **(Capability)**

**Probes:** Healthy foods; cost/convenience; cultural values and traditions.

Is your diet related decisions influenced by the advice or direction of another person? **(Opportunity - Social environment)**

**If yes:** What is that person’s role or relationship to you?

Describe how satisfied you are with the types and amounts of food you eat at home. **(Motivation)**

Describe some foods that you wish you would eat more of or more often. **(Motivation)    Probes:** Why?

Describe what types of foods you wish you would eat less of or less often. **(Motivation)    Probes:** Why?

Describe barriers that you face in making sure that you eat or drink what you think is ‘good’ or ‘healthy’. **(Capability/ opportunity)**

**Probes:** Time? Cost? Knowledge of what and how much to eat? Influence of other people?

What are the things that would make it helpful and motivate you to eat more healthily? **(Motivation/ capability)**

**Probes:** are there any strategies that you can use to help make sure you are getting the best diet? **(Capability)**

**Physical activity related questions**

Describe what physical activity means to you.

**Probes:** How do you decide if the amount or type of physical activity is good for health? **(Knowledge-perceptions)**

Describe how satisfied you are with the types and amounts of physical activity you engage in. **(Motivation)**

Do you ever worry you do not engage in enough physical activity? **(Motivation)**

Is your physical activity levels/types influenced by the advice or direction of another person? **(Opportunity - Social environment)**

**If yes:** What is that person’s role or relationship to you?

Describe the things you would like to change about how physically active you are.

**Probes:** Amount? Types of activity?

Describe barriers that you face in making sure that you are physically active. **(Capability/ opportunity)**

**Probes:** Time? Cost? Knowledge of how physically active you should be? Influence of other people? Household obligations?

What are the things that would make it helpful and motivate you to be physically active? **(Motivation/ capability)**

**Probes:** are there any strategies that you can use to help make sure you are engaging in the best physical activity? **(Capability)**

**Perceptions on future interventions**

So far, we mainly talked about eating healthily and being physically active. What else is important for you for a healthy life?

**Probes:** Are those things more important than eating healthily or doing sufficient physical activity?

How could we help/stimulate you to live healthily?

**Probes:** If we offered you guidance, what should that guidance look like? What is important to you regarding (lifestyle) guidance?

Where and when would you like to receive guidance for physical activity and diet?

**Probes:** Individually or in a group? Remotely or in person?

If we designed a program with weekly two-hour sessions at Alnahda where we do group exercise and share food recipes/healthy lifestyle tips, would you be interested?

**Probes:** would you be able to attend?
**Finally:** Offer the opportunity for participants to raise any further issues they feel we have not covered.
